# Potential applications of curcumin and its novel synthetic analogs and nanotechnology-based formulations in cancer prevention and therapy

**DOI:** 10.1186/1749-8546-6-31

**Published:** 2011-08-23

**Authors:** Murielle Mimeault, Surinder K Batra

**Affiliations:** 1 Department of Biochemistry and Molecular Biology, College of Medicine, Eppley Institute for Research in Cancer and Allied Diseases, University of Nebraska Medical Center, Omaha, NE 68198-5870, USA

## Abstract

Curcumin has attracted great attention in the therapeutic arsenal in clinical oncology due to its chemopreventive, antitumoral, radiosensibilizing and chemosensibilizing activities against various types of aggressive and recurrent cancers. These malignancies include leukemias, lymphomas, multiple myeloma, brain cancer, melanoma and skin, lung, prostate, breast, ovarian, liver, gastrointestinal, pancreatic and colorectal epithelial cancers. Curcumin mediates its anti-proliferative, anti-invasive and apoptotic effects on cancer cells, including cancer stem/progenitor cells and their progenies, through multiple molecular mechanisms. The oncogenic pathways inhibited by curcumin encompass the members of epidermal growth factor receptors (EGFR and erbB2), sonic hedgehog (SHH)/GLIs and Wnt/β-catenin and downstream signaling elements such as Akt, nuclear factor-kappa B (NF-κB) and signal transducers and activators of transcription (STATs). In counterbalance, the high metabolic instability and poor systemic bioavailability of curcumin limit its therapeutic efficacy in human. Of great therapeutic interest, the selective delivery of synthetic analogs or nanotechnology-based formulations of curcumin to tumors, alone or in combination with other anticancer drugs, may improve their chemopreventive and chemotherapeutic efficacies against cancer progression and relapse. Novel curcumin formulations may also be used to reverse drug resistance, eradicate the total cancer cell mass and improve the anticarcinogenic efficacy of the current anti-hormonal and chemotherapeutic treatments for patients with various aggressive and lethal cancers.

## Background

The deregulation and sustained activation of multiple tumorigenic pathways are typically implicated in cancer development and progression to locally advanced, aggressive and metastatic stages as well as in treatment resistance and disease relapse [1-5]. Consequently, the use of therapeutic agents acting on different deregulated gene products, alone or in combination therapy, may represent a potentially better strategy than the targeting of one specific oncogenic product to overcome treatment resistance and prevent cancer development and disease recurrence [1-5]. The non-toxic substance curcumin is the major bioactive ingredient extracted from the rhizome of the plant *Curcuma longa Linn*, also as known as turmeric [6,7]. Curcumin has been used as a dietary supplement as well as a therapeutic agent in Chinese medicine and other Asian medicines for centuries [6,7]. Recently, curcumin, which is a polyphenolic compound, has emerged worldwide as a potent therapeutic substance for treating diverse human diseases. Curcumin displays a wide range of pharmacological properties against various human disorders, such as metabolic and infectious diseases, diabetes, psoriasis, rheumatoid arthritis, atherosclerosis, Parkinson's and Alzheimer's diseases and cancer [6-14].

**Figure 1:**
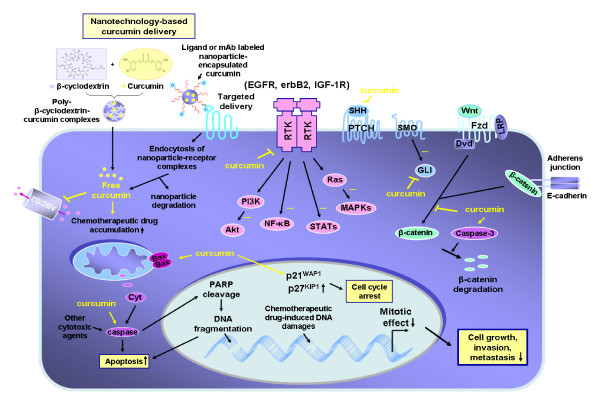
**Tumorigenic cascades initiated by different growth factors in cancer cells and the anticarcinogenic effects induced by dietary curcumin on the transduction signaling elements**. The inhibitory effect of curcumin on the expression and/or activity of EGFR, erbB2, IGF-1R, and their downstream signaling elements, sonic hedgehog (SHH/SMO/GLIs), Wnt/β-catenin and ATP-binding cassette multidrug transporters such as ABCG2 in cancer cells are indicated. Moreover, the enhanced expression of p21^WAP1 ^and p27^KIP1 ^cyclin-dependent kinase inhibitors and inhibition of mitotic effects induced by curcumin resulting in a cell cycle arrest and reduced expression levels of different gene products involved in the growth, invasion and metastasis of cancer cells as well as the activation by curcumin of mitochondrial factors and caspase pathway-induced apoptosis are also indicated. In addition, the scheme also shows novel nanotechnology-based curcumin delivery systems consisting of using either a poly(β-cyclodextrin)-curcumin complex formulation, or a polymeric micelle-encapsulated curcumin labeled with a ligand or monoclonal antibody (mAb) that specifically interacts with a receptor expressing by cancer cells for the selective targeting of curcumin are also illustrated.

**Figure 2:**
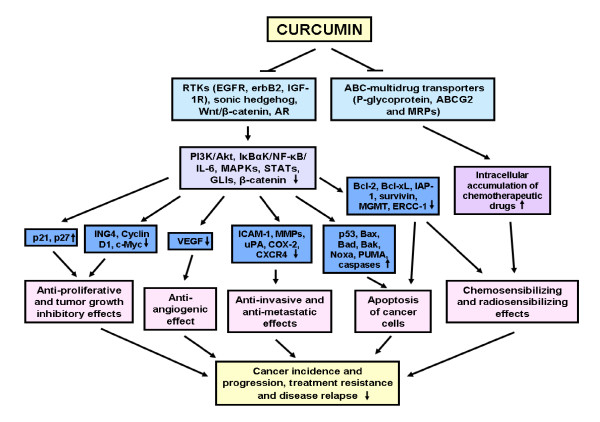
**Potential growth factor pathways, intracellular signal components and drug resistance-associated molecules modulated by curcumin involved in its chemopreventive and chemotherapeutic effects on cancer cells**. The scheme shows the inhibitory effects induced by curcumin on distinct oncogenic growth factor cascades and their multiple downstream intracellular signaling elements and ABC-multidrug transporters in cancer cells involved in the mediation of its cancer preventive and anticarcinogenic properties.

*In vitro *and *in vivo *studies have indicated that curcumin induces chemopreventive and chemotherapeutic effects against various types of human cancers. More specifically, curcumin exhibits anticarcinogenic effects on leukemias, lymphomas, multiple myeloma, brain cancer and melanoma as well as skin, cervix, lung, prostate, breast, ovarian, bladder, liver, gastrointestinal tract, pancreatic and colorectal epithelial cancers [2,9,15-36]. Curcumin displays strong anti-inflammatory, antioxidant, anti-aging, chemopreventive, antitumoral, anti-angiogenic, anti-metastatic, radiosensitizing and chemosensitizing effects in cancer cells in a concentration- and cell type-dependent manner (Figures 1 and 2) [2,7,9,10,22,37-39]. Of therapeutic interest, studies have indicated that curcumin as a single agent is safe and exhibits no major toxicity and only protects normal cells and organs at least in part by up-regulating the nuclear factor erythroid-derived-2 related factor 2 (Nrf2)-induced antioxidant gene products [8,38,40-46]. The anticarcinogenic effects induced by curcumin in cancer cells are mediated *via *the modulation of multiple oncogenic signaling transduction elements. Potential mechanisms of anticarcinogenic effects induced by curcumin in cancer cells include the down-regulation of the epidermal growth factor receptor (EGFR) family members (EGFR/erbB1 and erbB2/HER2), insulin-like growth factor type-1 receptor (IGF-1R), sonic hedgehog (SHH/GLIs) and Wnt/β-catenin and their downstream signaling effectors (Figures 1 and 2). The intracellular signaling transduction elements inhibited by curcumin include the signal transducers and activators of transcription (STATs), c-jun/activator protein-1 (AP-1), phosphatidylinositol-3'-kinase (PI3K)/Akt, nuclear factor-kappaB (NF-κB) and its targeted genes such as interleukin-6 (IL-6), cyclooxygenase-2 (COX-2) and matrix metalloproteinases (MMPs) (Figures 1 and 2) [2,9,17-21,24-30,47,48]. Other signaling components modulated through curcumin include the up-regulation of p21^WAP1 ^and p27^KIP1 ^cyclin-dependent kinase inhibitors and down-regulation of Bcl-2, Bcl-xL, survivin, induced myeloid leukemia cell differentiation protein-1 (Mcl-1) and glyoxalase 1 as well as the activation of Bax, Bad and caspase cascade-induced apoptosis (Figures 1 and 2) [2,9,15,17-21,24].

**Figure 3:**
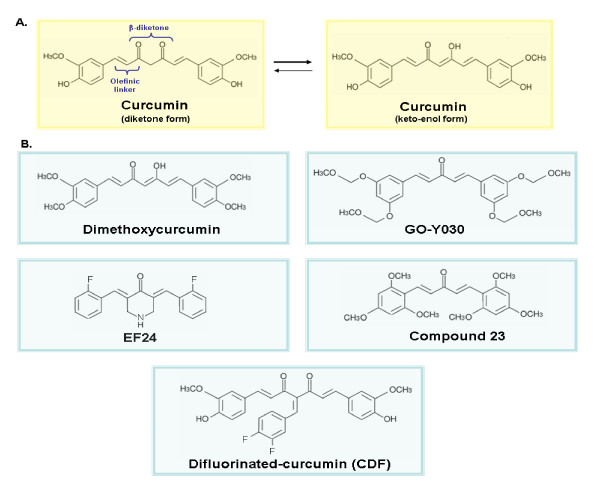
**Chemical structures of naturally occurring curcumin and its novel synthetic analogs**. The scheme shows (A) The diketone and keto-enol forms of curcumin. Curcumin exists as an equilibrium mixture of two tautomeric forms in solution. The enol structure of curcumin, which is stabilized by intramolecular H-bonding, is the most energetically stabilized and favored form; (B) chemical structures of novel synthetic analogs of dietary curcumin (dimethoxycurcumin, GO-Y039, EF24, compound 23 and difluorinated-curcumin "CDF") showing improved chemical stability and anticarcinogenic properties on different cancer cell lines.

In addition, some pre-clinical investigations have revealed that the administration of curcumin in the diet, alone or in combination with current therapeutic treatments, reduced cancer incidence, tumor development and progression to locally invasive and metastatic stages in animal models *in vivo *[2,16,34,49-54]. Importantly, curcumin and its derivatives can also inhibit proliferation and induce apoptosis on multidrug resistant cancer cells (*eg *cancer stem/progenitor cells with stem cell-like properties) by modulating the expression and/or activity of distinct survival pathways, ATP-binding cassette (ABC) multidrug transporters and micro RNAs (Figures 1 and 2) [15,55-70]. The data from trials with patients have also corroborated the safety profile and chemopreventive and chemotherapeutic effects of curcumin against diverse diseases and aggressive cancers in the clinical settings [9,37,69,71-81]. However, the therapeutic applications of curcumin in human are limited by its high metabolic instability as well as poor absorption and bioavailability. Synthetic analogs and formulations of curcumin have been developed, including its complexation with polymeric micelles or nanoparticle-based encapsulation that exhibit greater chemical stability, systemic bioavailability and antitumoral activities than naturally occurring curcumin (Figures 1 and 3) [7,24,82-101].

In this article, we review the most recent advances on the pharmacological characterization of the anticarcinogenic properties of curcumin and its novel synthetic analogs and nanotechnology-based formulations as well as the molecular mechanisms at the basis of the observed therapeutic effects induced by these agents.

### Search strategy

Literature search for this article was conducted in the MEDLINE/PubMed central database covering January 2000 to May 2011, with the term 'curcumin' alone and combined with other keywords including 'dietary agents', 'cancer', 'prostate cancer', 'brain cancer', 'pancreatic cancer', 'colorectal cancer', 'cancer stem cells', 'cancer prevention', 'cancer therapy', 'chemotherapy', 'structure-activity study', 'curcumin analogues'and 'curcumin formulation and nanotechnology'. Moreover, the term 'curcumin and cancer' was searched on two websites, namely http://www.google.com and http://www.clinicaltrials.gov. The relevant papers on chemopreventive and chemotherapeutic effects induced by curcumin or its derivatives, alone or in combination therapy, with an emphasis on brain, prostate, pancreatic and colorectal cancers were included in the review.

### Potential applications of curcumin in cancer prevention and therapy

Curcumin exhibits *in vitro *and *in vivo *chemopreventive and chemotherapeutic effects on various cancer cell types and animal models [2,7,16,26,34,50-54,102-114]. For instance, curcumin in the diet has been shown to prevent or counteract the inflammation- and carcinogen-promoted tumorigenesis *in vivo *in mouse models [16,49,50,53,112,114]. More specifically, it has been reported that curcumin triggered the apoptosis on the murine K-Ras-induced lung adenocarcinoma cell line (LLR-10 and LKR-13) [112]. Moreover, 1% curcumin in the diet suppressed the non-typeable *Hemophilius influenzae *(NTHi)-induced chronic airway inflammation and lung cancer progression in mice through anti-inflammatory and anti-tumoral effects [112]. In the same manner, a topical application of curcumin also prevented the formation of benzo[a]pyrene-DNA adducts and its tumorigenic activity in epidermis in CD-1 mice [49]. A topical application of curcumin was also effective at inhibiting the skin tumor promotion mediated by 12-O-tetradecanoylphorbol-13-acetate (TPA) in 7,12-dimethylbenz[a]anthracene-initiated mouse skin [49]. It has also been observed that the administration of 0.5-2.0% commercial grade curcumin (77% curcumin, 17% demethoxycurcumin and 3% bisdemethoxycurcumin) in the diet inhibited benzo(a)pyrene-induced forestomach tumorigenesis in A/J mice, N-ethyl-N'-nitro-N-nitrosoguanidine-induced duodenal tumorigenesis in C57BL/6 mice and azoxymethane (AOM)-induced colon tumorigenesis in CF-1 mice or F344 rats [16,53].

In addition, curcumin has also been shown to suppress proliferation while it induced apoptosis and radiosensibilizing and chemosensibilizing effects on diverse human cancer cell types, including leukemia and lymphoma cells, multiple myeloma cells and brain, melanoma and epithelial cancer cells (Figures 1 and 2) [17,25,26,34,39,102,110,115-118]. The cytotoxic effects of curcumin were mediated by down-regulating the sustained activation of PI3K/Akt and/or IκBα kinase (IκBαK) and nuclear translocation of NF-κB and STATs induced by growth factors (Figures 1 and 2) [17,25,26,34,39,102,110,115-118]. For instance, it has been observed that curcumin down-regulated the constitutive activation of IκBα kinase-induced NF-κB and the expression of these target genes, including IL-6, cyclin D1, Bcl-2 and Bcl-xL in human multiple myeloma cells [26]. The curcumin treatment of multiple myeloma cells was also effective at suppressing the proliferation, inducing apoptosis and improving the sensitivity of these cancer cells to the cytotoxic effects induced by chemotherapeutic drugs, vincristine and melphalan [26]. Moreover, curcumin induced antiproliferative and apoptotic effects on human A375, C32, G-361 and WM 266 melanoma cell lines, all of which have B-Raf mutations, B16-R melanoma cells resistant to doxorubicin and novel mouse melanoma cells, whereas curcumin induced no cytotoxic effect on normal melanocytes [33,119-122]. The cytotoxic effects of curcumin on these melanoma cell lines were mediated in part through the down-regulation of the constitutive activation of IκBα kinase-induced NF-κB in a manner independent of the B-Raf/MEK/ERK and Akt pathways [33,119-122]. It has been noticed that a combination of low doses of curcumin plus tamoxifen resulted in a synergistic induction of apoptosis and autophagy in chemoresistant melanoma cells and the silencing of multidrug resistance transporter ABCA1 in highly tumorigenic and metastatic human M14 melanoma cells, which are resistant to curcumin treatment, restored their sensibility to curcumin [122,123]. Importantly, the results from *in vivo *studies consisting of the intraperitoneal injection of curcumin at doses of 50 and 100 mg/kg every 2 days, respectively have also indicated that this dietary compound inhibited the tumor growth and spontaneous metastasis of B16BL6 melanoma cells in mice at least in part by down-regulating the expression at the transcriptional level of an oncogenic product, phosphatase of regenerating liver-3 (PRL-3) [34]. Furthermore, curcumin also reduced the invasion and strongly induced apoptosis in the human estrogen receptor-α (ER-α)-negative and aggressive MDA-MB-231 breast cancer cell line *in vitro *concomitant with a down-regulation of the NF-κB survival pathway and expression levels of inflammatory cytokines CXCL1 and CXCL2, CXCR4 and MMP [35,36]. Moreover, 1% curcumin in the diet decreased the incidence of lung metastases derived from MDA-MB-231 cells injected into the heart of immunodeficient mice [36].

Importantly, despite the fact that curcumin may act as a cytotoxic, chemosensitizing and radiosensitizing agent in cancer cells, it can also protect normal cells and organs such as brain, intestine, liver, kidney, oral mucosa, heart and spleen against oxidative stress and chemotherapy- and radiotherapy-induced toxicity [38,40-46,73,124]. The protective effects of curcumin appear to be mediated through its ability to directly scavenge free radicals or indirectly by up-regulating the endogenous cellular antioxidant mechanisms including the activation of cytoprotective Nrf2-induced target genes [8,38,40-46,124]. In fact, Nrf2 acts as a transcriptional activator of the antioxidant responsive element (ARE)-mediated gene expression, including phase II detoxification and antioxidant stress enzymes such as hemeoxygenase-1, glutathione peroxidase, modulatory subunit of gamma-glutamyl-cysteine ligase, which is involved in glutathione synthesis, and NAD(P)H:quinone oxidoreductase 1 [38,40-46]. Thus, the modulation of these gene products by curcumin may contribute in part to its antioxidant and cytoprotectrive effects in normal cells including its neuroprotective activity [38,40-46].

Together, these observations suggest that curcumin may counteract the development of a variety of cancers and overcome resistance to current radiotherapy and chemotherapy that may be promoted by oxidative stress and sustained activation of the survival pathways such as Akt and NF-κB without major toxicity on normal cells (Figures 1 and 2). We report in a more detailed manner the recent advances on *in vitro *and *in vivo *studies of the chemopreventive and chemotherapeutic effects of curcumin that have been performed on brain, prostate, pancreatic and colorectal cancers as well as the characterization of the pharmacological properties of novel curcumin analogs and formulations with improved chemical stability and anticarcinogenic properties.

#### Brain cancer

Medulloblastomas and malignant gliomas are among the most aggressive primary brain tumors that frequently occur in children and adults respectively [125-128]. Importantly, curcumin has been shown to suppress the proliferation, trigger cell cycle arrest at the G_2_/M phase and induce apoptosis in medulloblastoma and glioma cells *in vitro *and in an animal model *in vivo *[129-140]. More specifically, curcumin induced the anti-proliferative, anti-migratory and apoptotic effects on medulloblastoma cells *via *the down-regulation of the expression levels of the SHH ligand and the GLI-1 transcriptional effector of the hedgehog cascade, β-catenin, the phosphorylated forms of Akt and NF-κB as well as their downstream targets such as c-Myc, N-Myc, cyclin D1 and anti-apoptotic factors Bcl-2 and Bcl-xL (Figures 1 and 2) [129,130]. It has been noticed that the curcumin-resistant medulloblastoma cells, which exhibited no decrease in the levels of SHH and Bcl-2 levels could be sensitized to curcumin by a co-treatment with SMO antagonist, cyclopamine [129]. The apoptotic effect of curcumin was also enhanced by another dietary substance, namely piperine, the main alkaloid from black pepper that acts as an enhancer of curcumin bioavailability in humans [129]. Moreover, curcumin was also effective at improving the cytotoxic effects induced by cisplatin and γ-rays *via *the down-regulation of the anti-apoptotic factor Bcl-2 in medulloblastoma cells [129].

In addition, several studies have indicated that curcumin can induce the antiproliferative, apoptotic, radiosensibilizing and chemosensibilizing effects on glioma cells *via *the up-regulation of p53, p21^WAF1 ^and the inhibitor of growth 4 (ING4), inhibition of NF-κB and AP-1 transcriptional activities and stimulation of the caspase cascade [132-135,138-140]. For instance, curcumin induced a histone hypoacetylation in glioma cells and apoptotic cell death through a poly (ADP-ribose) polymerase (PARP)- and caspase 3-mediated pathway while it promoted the neurogenesis in neural progenitor cells (Figure 1) [132]. Moreover, curcumin was also effective at attenuating the cell viability of human (T98G, U87MG and T67) and rat C6 glioma cell lines *via *the inhibition of Akt/NF-κB and c-Jun N-terminal kinase (JNK)/AP-1 signaling pathways [133]. Of clinical interest, curcumin has also been observed to sensitize glioma cells to radiation and several current chemotherapeutic drugs, including cisplatin, etoposide, camptothecin and doxorubicin through a reduced expression of Bcl-2 and the inhibitor of apoptosis proteins (IAPs) as well as DNA repair enzymes such as O6-methylguanine-DNA methyltransferase (MGMT), DNA-dependent protein kinase, Ku70, Ku80 and excision repair cross-complementing rodent repair deficiency, complementation group 1 (ERCC-1) [133].

Together, these results suggest that curcumin or its derivatives could be used as adjuvant treatment for improving the anticarcinogenic efficacy of current radiation therapy and chemotherapy against locally advanced, disseminated and recurrent medulloblastomas and gliomas, which retain lethal with the current treatment options.

#### Prostate cancer

Accumulating experimental lines of evidence have indicated that curcumin is effective in counteracting prostate cancer initiation and progression to locally invasive, androgen-independent (AI) and metastatic disease stages [7,16,51,103-109,141]. It has been shown that curcumin can induce the antiproliferative, anti-invasive, antiangiogenic and apoptotic effects on human AI PcBra1 cells from localized prostate cancer and metastatic and androgen-dependent (AD) LNCaP and AI C4-2B, DU145 and PC3 prostate cancer cells *in vitro *and *in vivo*, without any toxic effect on normal prostate epithelial cells (PrECs) [7,51,103-109,141]. More specifically, curcumin may mediate growth inhibitory and apoptotic effects in AD and AI prostate cancer cells by down-regulating the expression and/or activity of diverse oncogenic and survival signaling components, including EGFR, erbB2, hedgehog, androgen receptor (AR) and PI3K/Akt, NF-κB, Bcl-2, Bcl-xL and TMPRSS2-ERG fusion protein (Figures 1 and 2) [107,108]. Curcumin can also cause DNA damage and apoptotic/necrotic death of prostate cancer cells by up-regulating diverse pro-apoptotic factors such as the p53 tumor suppressor protein, Bax, Bak, Noxa, p53 up-regulated modulator of apoptosis (PUMA) and/or BCL-2-like 11 (Bim) [107,108]. For instance, it has been reported that curcumin inhibited the growth and triggered the apoptosis of AD LNCaP and AI PC3 cells *in vitro *by down-regulating the expression levels and intrinsic activities of EGFR and its downstream signaling elements, including PI3K/Akt and NF-κB (Figures 1 and 2) [141,142]. Moreover, curcumin effectively inhibited the SHH hedgehog ligand-stimulated growth of the mouse prostate cancer cell line derived from transgenic adenocarcinoma of the mouse prostate (TRAMP) designated as TRAMP-C2, LNCaP and PC3 cells at least in part, by inhibiting the hedgehog cascade and GLI-1 expression [51]. Additionally, it has also been reported that the treatment of PC3 cells with curcumin *in vitro *reduced the expression level and activity of CC motif ligand 2 (CCL2) and MMP-9 proteolytic activity, thereby suppressing the cell adhesion, motility and invasion [109].

Of particular interest, a combination of low doses of curcumin and other dietary phytochemicals or anticancer drugs also induced greater anticarcinogenic effects on prostate cancer cells than individual agents [51,52]. For instance, a treatment of 8-week old TRAMP mice with a diet supplemented with 2% curcumin or 0.05% β-phenyethylisothiocyanate (PEITC), or a combination of 1% curcumin plus 0.025% PEITC for a period of 10 or 16 weeks significantly inhibited the incidence of the formation of high-grade prostatic intraepithelial neoplasias and prostate cancer development, at least in part, by down-regulating the Akt pathway [51,52]. The intraperitoneal injection of a combination of 3 μmol curcumin plus 2.5 μmol PEITC was also more effective than a higher dose of 6 μmol curcumin or 5 μmol PEITC alone at inhibiting the tumor growth of PC3 cell xenografts in immunodeficient mice by inhibiting Akt and NF-κB [51,52]. Moreover, curcumin also sensitized LNCaP and PC3 cells *in vitro *and LNCaP xenografts to tumor necrosis factor-related apoptosis-inducing ligand (TRAIL)-induced apoptosis by up-regulating TRAIL-R1 and R2 (DR4 and DR5), Bax, Bak, p21^WAF1 ^and p27^KIP1 ^and down-regulating pAkt-induced NF-κB and its targeted gene products such as cyclin D1, vascular endothelial growth factor (VEGF), urokinase-like plasminogen activator (uPA), MMP-2 and MMP-9 [143-145]. More specifically, a combination of curcumin (30 mg/kg, three days per week) administered by oral injection plus TRAIL (15 mg/kg, four times during first three weeks) administrated by intravenous injection resulted in greater tumor growth inhibitory and anti-angiogenic effects on LNCaP cells subcutaneously implanted in nude mice as compared to individual agents [143-145].

Together, these data support the therapeutic interest of using curcumin or its derivatives, alone or in combination with other dietary substances, to improve the efficacy of the current anti-hormonal and chemotherapeutic treatments against locally advanced, hormone-refractory and metastatic prostate cancers.

#### Pancreatic cancer

Pancreatic cancer is a highly lethal disease with a poor long-term overall five-year survival rate of less than 5% for patients diagnosed with locally advanced and metastatic disease stages [146-148]. The poor prognosis of patients is in part due to the early occurrence of metastatic spread and the development of intrinsic and acquired resistance by cancer cells during drug treatment [146,147,149,150]. This lack of efficacy of the current clinical therapies by surgical resection, radiotherapy and/or gemcitabine-based chemotherapies against aggressive and metastatic pancreatic cancers underlines the urgent need to validate novel therapeutic agents for overcoming treatment resistance. Importantly, curcumin has been shown to induce the anti-proliferative, apoptotic, anti-angiogenic and chemosensibilizing effects on diverse pancreatic cancer cells *in vitro *and *in vivo *[27,69,70,151-159]. The anticarcinogenic effects of curcumin were mediated through the down-regulation of the expression and/or activity of distinct signaling elements, including EGFR, STAT-3, NF-κB and its targeted genes, multidrug transporters such as multidrug resistance-associated protein 5 (MRP5), and modulation of the expression levels of different micro RNAs [27,69,70,151-159]. For instance, curcumin inhibited the proliferation of Panc28 and L3.6pL pancreatic cancer cells *in vitro *by down-regulating NF-kB-dependent gene transactivation and Sp1, Sp2 and Sp3 transcription factors, which are overexpressed in pancreatic cancers [153]. The intraperitoneal injection of curcumin in corn oil (100 mg/kg/day, each 2^nd ^day for 18 days) also suppressed the tumor growth of L3.6pL cell xenografted in nude mice [153]. Moreover, curcumin potentiated the anti-proliferative and apoptotic effects induced by gemcitabine, a first-line chemotherapeutic drug, on BxPC-3, Panc-1 and MiaPaCa-2 pancreatic cancer cell lines *in vitro *[27,154]. A combination of curcumin (1 g/kg, once daily), administered orally plus an intraperitoneal injection of gemcitabine (25 mg/kg, twice weekly) was more effective than single agents at inducing the tumor growth inhibitory and anti-angiogenic effects in a pancreatic tumor model derived from MiaPaCa-2 pancreatic cancer cells orthotopically implanted in nude mice [27,154]. The chemosensibilizing effects of curcumin were mediated at least in part *via *the inhibition of STAT-3 and NF-kB-regulated gene products such as cyclin D1, c-Myc, Bcl-2, Bcl-xL, cellular IAP-1, COX-2, MMPs and VEGF in pancreatic cancer cells (Figures 1 and 2) [27,154]. Of particular interest, it has also been observed that a combination of low doses of curcumin and other dietary agents (isoflavone, resveratrol and epigallocatechin-3-galate), COX-2 inhibitor (celecoxib) or an omega-3 fatty acid (docosahexaenoic acid) induced synergistic growth inhibitory and apoptotic effects on pancreatic cancer cells *in vitro *and *in vivo *[160-162].

Together, these data support the therapeutic interest of using low doses of curcumin or its derivatives in combination therapy with other cytotoxic agents acting on multiple molecular targets as chemopreventive treatment in the diet or to improve the efficacy of the current gemcitabine-based chemotherapeutic regimens against locally advanced, metastatic and recurrent pancreatic cancers.

#### Colorectal cancer

The loss of function by inactivating mutations in the *adenomatous polyposis coli *(APC) or axis inhibition protein (axin) tumor suppressor proteins or activating mutations in β-catenin concomitant with the activation of the Wnt signaling pathway and nuclear accumulation of β-catenin frequently occurs during gastrointestinal cancers, including colorectal cancer initiation and progression, and leads to an enhanced expression of diverse oncogenic products (Figures 1 and 2) [163-165]. Moreover, the activation of several tumorigenic signaling elements, such as EGFR, erbB2, mucin 1, Ras, PKC-βII and orphan nuclear receptor peroxisome proliferator-activator receptor-γ (PPAR-γ), can promote the release of β-catenin from the adherens junction complexes with E-cadherin and/or its nuclear translocation (Figure 1) [163,166]. Thus, the association of nuclear β-catenin with the T cell factor (TCF)/lymphoid enhancer factor (LEF) family of transcription factors may up-regulate the expression of several gene products such as c-Myc, cyclin D1, gastrin, COX-2, MMP-7, uPA receptor, CD44 and P-glycoprotein that are involved in colorectal cancer development and treatment resistance (Figure 2) [163]. Importantly, it has been reported that the administration of 0.6% curcumin in the diet prevented the progression of colorectal cancer associated with colitis in C57BL/6 mice by inhibiting the translocation of β-catenin from adherens junction complexes to the cytoplasm and nucleus and reducing the levels of diverse proinflammatory cytokines, inducible nitric oxide synthase (iNOS) and COX-2 as compared to untreated mice (Figure 1) [167]. Moreover, the administration of 0.2% or 0.5% curcumin in the diet, approximately equal to 300 and 750 mg/kg curcumin per day respectively, commencing one week postweaning in *APC^-/+ ^*mice, also reduced the incidence of adenocarcinoma formation as compared to untreated *APC^-/+ ^*mice [2,54]. In the same manner, a treatment with curcumin (250 mg/kg body weight), alone or in combination with dasatinib (10 mg/kg body weight), for five consecutive days a week for 4 weeks, was also effective at inducing tumor regression in a familial *APC^-/+^*mouse model as compared to untreated *APC^-/+ ^*mice [2,54]. Additionally, curcumin was effective at inhibiting tumor growth, invasion and *in vivo *metastasis of human RKO and HCT-116 colon cancer cells (wild-type *p53^+/+^*) in the chicken-embryo-metastasis assay in part by down-regulating the transcriptional expression of micro RNA-21 and up-regulating the programmed cell death protein-4 (PDCD4), which is a target of micro RNA-21 [168].

In addition, curcumin has also been reported to cause p53- and p21-independent G_2_/M phase arrest, caspase-3-mediated cleavage of β-catenin, decreased transactivation of gene products such as c-Myc induced by β-catenin/TCF/LEF complex, and an enhanced rate of apoptosis in HCT-116 (*p53^+/+^*), HCT-116 (*p53^-/-^*) and HCT-116 (*p21^-/-^*) colon cancer cell lines (Figure 1) [169]. A combination of curcumin with another dietary resveratrol, pan-erbB inhibitor (EGF-R related protein, ERRP), Src inhibitor dasatinib, 5-fluorouracil and/or oxaliplatin also induced greater anti-proliferative, anti-invasive and/or apoptotic effects on diverse colorectal cancer cell lines than individual drugs *in vitro *and *in vivo *[2,170-172]. The therapeutic effects of these combination therapies were mediated through a reduction of the activated EGFR, erbB2, IGF-1R and Src phosphorylated forms and decreased expression levels and activities of extracellular signal-regulated kinases (ERKs), pAkt, NF-κB, Bcl-xL and/or COX-2 and caspase activation (Figures 1 and 2) [2,171,172]. For instance, it has been observed that a combination of curcumin with the current chemotherapeutic drugs, namely 5-fluorouracil and/or oxaliplatin used for treating patients with advanced colorectal cancer, synergistically inhibited the growth of colon cancer cells *in vitro *[172]. A combination of curcumin plus diverse chemotherapeutic drugs such as cisplatin, doxorubicin, danorubicin and vinscritin was also accompanied by an enhanced intracellular accumulation and improved cytotoxic effects of drugs on colorectal cancer cells [173]. Importantly, a combined treatment of curcumin given orally (1 g/kg once daily) with capecitabine given by gavage (60 mg/kg twice weekly) was also more effective than single agents at inhibiting tumor growth, angiogenesis and metastases at ascites and distant tissues such as the liver, intestine, lung, rectum and spleen of HCT-116 colon cancer cells orthotopically implanted in nude mice [116]. The sensibilizing effects of curcumin on the antitumoral and anti-metastatic properties of capecitabine were mediated through a decreased expression of NF-kB-regulated gene products such as c-Myc, Bcl-2, Bcl-xL, cIAP-1, COX-2, intercellular adhesion molecule 1 (ICAM-1), MMP-9, CXC chemokine receptor 4 (CXCR4) and VEGF (Figure 2) [116].

Thus, it appears that curcumin and its derivatives are promising agents to target Wnt/β-catenin and NF-kB in colorectal cancer cells, thereby counteracting cancer initiation and progression and improving the efficacy of the current chemotherapeutic treatments. Consistent with this, the results from some recent investigations have revealed that curcumin and its derivatives are also effective at inducing the cytotoxic effects on chemoresistant cancer cells, including cancer stem/progenitor cells from colorectal cancer cell lines and other cancer cell types.

#### Cytotoxic effects of curcumin on cancer stem/progenitor cells

A growing body of experimental evidence has revealed that self-renewing and tumorigenic cancer stem/progenitor cells endowed with stem cell-like properties, also designated as cancer- and metastasis-initiating cells, can provide critical functions for cancer initiation and progression, treatment resistance and disease recurrence [4,174]. Of great therapeutic interest, curcumin has been reported to inhibit the clonogenecity and induce the anti-proliferative and apoptotic effects on drug-resistant and sphere-forming cancer cells expressing stem cell-like markers as well as reverse the chemoresistance and improve the cytotoxic effects induced by diverse chemotherapeutic drugs on these immature cancer cells [59-61]. For instance, curcumin, alone or in combination with piperine, inhibited the mammosphere formation and decreased the number of aldehyde dehydrogenase-expressing cells detected in non-malignant and malignant MCF-7 and SUM159 breast cells through the inhibition of Wnt signaling cascade [59]. This suggests the possibility of using a dietary curcumin supplement as a chemopreventive agent for breast cancer. Moreover, the treatment of HCT-116 or HT-29 colon cancer cells with 5-fluorouracil and oxaliplatin also resulted in an enrichment of cancer cells with stem cell-like phenotypes as evidenced by an increased proportion of cancer cell fractions expressing high levels of CD133, CD44, CD166 and/or EGFR levels [60]. By contrast, curcumin, alone or in combination with 5-fluorouracil and oxaliplatin, induced a marked reduction in cancer stem cell-like cells, as indicated by a decrease in the expression levels of CD133, CD44, CD166 and EGFR as well as their ability to impair the colonosphere formation *in vitro *of chemosurviving HCT-116 or HT-29 colon cancer cells [60].

On the other hand, among the other methods frequently used for the enrichment of a small population of cancer stem/progenitor cells from cancer cell lines, there is the Hoechst dye efflux technique that is particularly useful when the stem cell-like markers are not well-established [1,175,176]. In fact, the analysis of the total cancer cell mass by Hoechst 33342 dye efflux technique can detect a small fraction of cancer cells with stem cell-like properties designated as a side population (SP) that possesses a higher ability to actively efflux the fluorescent DNA-binding dye, Hoechst 33342 than the non-SP cell fraction due to its elevated expression levels of ATP-binding cassette (ABC) multidrug efflux pumps [1,175,176]. In the regard, numerous studies have revealed that the SP cell fraction detected in various cancer cell lines, including leukemia, brain cancer, melanoma and epithelial cancers possesses the stem cell-like properties [1,175,176]. Curcumin and its major metabolite, namely tetrahydrocurcumin, have also been reported to down-regulate the expression and/or activity of multiple ABC multidrug transporters, including ABCG2, multidrug resistance 1 (MDR-1) encoding P-glycoprotein (ABCB1) and multidrug resistance protein-1 (MRP-1; ABCC1) in parental cancer cell lines and their derivatives that are resistant to multiple drugs, the SP cell fraction and patient leukemic cells *in vitro *and in mice *in vivo *[61-68]. Thus, curcumin can improve the bioavailability and intracellular accumulation of diverse chemotherapeutic drugs, reverse the chemoresistance and act in cooperation with the other drugs to induce greater cytotoxic effects (Figures 1 and 2). For instance, it has been reported that the treatment of rat C6 glioma cells with curcumin for 3-10 days or during the Hoechst 33342 dye exclusion assay, resulted in a significant decrease in the number C6 glioma cells detected in the SP cell fraction by flow cytometry, suggesting that curcumin can inhibit multidrug resistance transporters in stem cell-like glioma cells [61].

Additional studies are, however, required to corroborate these results on the cancer stem/progenitor cell subpopulations isolated cancer cell lines and those detected in the patients in clinical settings.

### Clinical trials of curcumin

The results from phase I/II clinical trials including the dose-escalation studies with pure curcumin or curcumin extract have indicated that oral administration of this dietary compound as single agent is generally well-tolerated, non- or little toxic and induced the chemopreventive and chemotherapeutic effects on some types of diseases and aggressive cancers [69,71-81]. More specifically, it has been reported that the administration of curcumin as single agent at dose levels of up to 100-8000 mg/day was associated with no discernible or only minimal toxicity while a highest dose of 12,000 mg/day was not acceptable to some patients because of the large amount of the curcumin capsules necessary to reach this high dose [69,71-74,76-81,177]. The potential toxicity and side effects that have been observed with the use of curcumin as a single agent given orally to the patients include mild diarrhea and nausea, headache, rash and yellow stool [71-73,76,79]. Despite these clinical data suggesting that oral curcumin as single agent is little toxic, further studies using escalating dose levels of curcumin on a greater number of patients are necessary to confirm its tolerability and safety profile after long-term use, and more particularly in combination therapies with other drugs. In this regard, we are reporting accumulating lines of evidence that have indicated the feasibility and safety to use the curcumin, alone or in combination with other chemotherapeutic agent, in cancer prevention and therapies.

#### Clinical investigations of the chemopreventive and chemotherapeutic effects of curcumin

Recent studies have indicated that curcumin exhibits chemopreventive and chemotherapeutic effects on some patients with pre-malignant lesions or different cancers including oral, breast, prostate, pancreatic and colorectal cancers (Additional file 1) [71-73,76-81,177,178]. More particularly, the data from a phase I dose-escalation study performed with 25 patients at high risk of developing cancer or with precancerous lesions and consisting of the administration of 500-12,000 mg/day of oral curcumin for 3 months have indicated that curcumin was well-tolerated, non-toxic at doses of 8000 mg or lower and induced a histolological improvement of precancerous lesions in some patients [71]. A histolological improvement has been observed in one patient with recently resected bladder cancer, two patients with oral leucoplakia, one patient with intestinal metaplasia of the stomach, one patient with uterine cervical intraepithelial neoplasm and two patients with Bowen's disease [71]. Moreover, the results from a pilot study on 15 patients with advanced colorectal cancer refractory to standard chemotherapies have also revealed that five patients had stable disease after treatment with 2200 mg daily of oral curcuma extract equivalent to 180 mg of curcumin for 2-4 months [72]. The data from a phase II trial carried out with 21 evaluable pancreatic cancer patients, which consisted of a treatment with 8000 mg of curcumin by month daily until disease progression, with restaging every two months, have also indicated that curcumin was detectable in the peripheral circulation under glucuronide and sulfate conjugate forms [77]. These results suggest that a high rate of metabolic transformation and poor tissue distribution of curcumin may occur in cancer patients. Although curcumin is highly metabolic instable with poor bioavailability, two pancreatic cancer patients showed clinical biological response to curcumin according to Response Evaluation Criteria in Solid Tumors Group (RECIST) [77,179]. More specifically, one patient had ongoing stable disease for more than 18 months and another additional patient had a brief but marked tumor regression (73%) while no toxicity was observed [77].

Other clinical trials have also confirmed the safety and feasibility to use curcumin in combination therapy with current chemotherapeutic treatments (Additional file 1) [81,177,178]. For instance, the results from a phase I/II study on 21 patients with disease progression with gemcitabine-based chemotherapy have indicated that the median overall survival time of the patients after a treatment with curcumin plus gemcitabine or gemcitabine/S-1 combination was 161 days and 1-year survival rate of 19% (95% confidence interval) (Additional file 1) [81]. Despite no partial or complete response of pancreatic cancer patients was noted in this study, five patients showed a stable disease according to RECIST criteria [81,179]. Moreover, the results from another study on 17 patients with advanced pancreatic cancer, who were treated with a dose of 8000 mg of curcumin by month daily plus gemcitabine, have indicated that the time to tumor progression was 1-12 months (median 2 1/2), and overall survival was 1-24 months (median 5) [178]. Among 11 evaluable patients in this study, one patient had a partial response, four had stable disease and six showed tumor progression [178]. In addition, the data from a phase I trial of dose-escalating curcumin that was given orally plus docetaxel administrated as intravenous infusion, which was carried out on 14 patients with advanced and metastatic breast cancer, have also indicated that five patients showed a partial tumor response and three patients had a stable disease with this combination therapy according to RECIST criteria (Additional file 1) [177,179]. The grades 3-4 hematological toxicity such as neutropenia and leucopenia was observed after docetaxel treatment in most patients in this study including a grade 4 neutropenia with a dose-limiting toxicity (DLT) as well as two grade 3 diarrhea with DLTs in two patients, grade 1 mucositis of oral cavity in three patients, grade 1 hand-foot syndrome in two patients and dermatological and lymphatic toxicity in four patients [177]. The observations of two DLTs, including one grade 4 neutropenia and one grade 3 diarrhea at a dose of 8000 mg/day of curcumin, combined with the poor acceptability of this high dose of curcumin (16 capsules/day) by two patients has led to define the maximal tolerated dose (MTD) of the curcumin at 8000 mg/day for this combination therapy [177].

Additional clinical trials are however necessary to more precisely establish the toxicity and antitumoral effects induced by combined docetaxel plus curcumin *versus *the docetaxel or curcumin alone in a greater number of the locally advanced and metastatic breast cancer patients. Based on these encouraging results, phase I/II/III clinical trials are now ongoing to investigate the antitumoral activity of curcumin, alone or in combination with the current chemotherapeutic drugs, in patients diagnosed with a variety of cancers, including multiple myeloma and non-small cell lung, advanced breast, pancreatic and colorectal cancers. Thus, the results from these additional clinical trials with curcumin or its derivatives should confirm their pharmacodynamic and pharmacokinetic profiles and therapeutic efficacy, alone or in combination therapy, for treating patients with a wide range of aggressive and recurrent cancers.

Together, these observations indicate that curcumin is generally well-tolerated and without major toxicity and displays anticarcinogenic activity on different cancer cell types and some cancer patients without secondary effects on normal tissues. This natural dietary compound, however, exhibits a poor absorption and metabolic instability which may limit its delivery and biological activity in the tumoral tissues when administrated orally. In this regard, we discuss here novel strategies that have been elaborated to optimize the formulations and mode of administration of curcumin for improving its bioavailability, selective delivery to tumoral tissues and anticarcinogenic effects in cancer patients.

### New strategies for improving the physical and metabolic stability, bioavailability and antitumoral effects of curcumin

Although free curcumin [1,7-bis(4-hydroxy-3-methoxyphenyl)-1,6-heptadiene-3,5-dione] (also designated as diferuloylmethane, Figure 3) possesses multiple therapeutic effects, the major disadvantages associated with its oral administration are its high physical and metabolic instability and poor aqueous solubility at neutral and basic pH values limiting its systemic bioavailability and efficacy under physiological conditions [77,78,180-182]. As mentioned previously, the data from preclinical and clinical studies have indicated that curcumin displays chemopreventive and chemotherapeutic effects and is safe even at high doses in animal models and humans. In counterbalance, curcumin typically exhibits a high metabolic instability, poor tissue distribution and systemic bioavailability. These chemical features of curcumin limit its clinical applications to treat gastrointestinal tract malignancies that are exposed to unmetabolized and active forms of curcumin [11,77,78,183,184]. The main reasons that may contribute to the low plasma level, limited tissue distribution and decreased therapeutic efficacy of curcumin are as follows: (1) poor absorption in the gastrointestinal tract across the gut, (2) extensive metabolism through oxidation, reduction, glucuronidation and sulfation, yielding less active metabolites, and (3) rapid elimination from the body [78,181-185]. The major metabolic products of curcumin detected in hepatocytes in suspension *in vitro *as well as *in vivo *after curcumin treatment in rodent and human comprise dihydrocurcumin, tetrahydrocurcumin, hexahydrocurcumin, hexahydrocurcuminol and their glucuronide and sulfate conjugates [72,78,181,183]. Of great clinical interest, it has been observed that the combination of 2000 mg/day curcumin with an inhibitor of hepatic and intestinal glucuronidation, piperine (20 mg/kg) resulted in higher curcumin concentrations in serum and substantially improved bioavailability of curcumin in healthy human volunteers [186]. Moreover, several synthetic analogs of curcumin have been designed and shown to exhibit a greater metabolic stability and biological activity than those of curcumin itself and are without increased toxicity (Figure 3) [7,32,82-88,185,187-212]. A variety of experimental strategies and carrier systems have also been developed to improve the selective and sustained delivery of curcumin into cancer cells. These strategies include the use of curcumin phospholipid complexes, the inclusion of curcumin in liposomes/lipidic micelles or the curcumin encapsulation in diverse polymeric nanoparticle-based formulations which may be unconjugated or conjugated to a ligand or antibody that specifically targets the cancer cell receptor or epitopes (Figure 1) [24,89-101,213,214].

#### New synthetic analogs of curcumin

A potential strategy to enhance the anticarcinogenic efficacy and overcome the high physical and metabolic instability and poor bioavailability of curcumin may be the use of the synthetic chemical analogs of this natural dietary compound endowed with improved physicochemical and pharmacological properties. Structure-activity studies based on the tautomeric forms of naturally occurring curcumin have led to the development of some synthetic analogs endowed with a better chemical stability and showing more potent anti-inflammatory, anti-oxidant, anti-carcinogenic and/or anti-angiogenic effects on diverse human cancer cell lines than curcumin (Figure 3) [7,32,82-88,185,187-212,215,216]. For instance, it has also been observed that the protection of the 4-OH groups of curcumin through methylation yielded a dimethoxycurcumin analog exhibiting an enhanced metabolic stability *in vitro *and *in vivo *as well as greater anti-proliferative and apoptotic effects on HCT-116 colorectal cancer cells and anti-proliferative activity on breast and prostate cancer cell lines as compared to curcumin (Figure 3) [185,187]. Moreover, another synthetic analog of curcumin with a pyrazole ring mimicking the enol form of curcumin, designated as compound 12, was three to four-folds more effective than curcumin at inducing the anti-proliferative effects and showed the anti-angiogenic and anti-androgenic activities on breast and prostate cancer cell lines [187,189,192,204].

In addition, other synthetic analogs of curcumin also include FLLL11, FLLL12, FLLL32 and GO-Y030 (Figure 3), which are more potent than curcumin at inhibiting the growth, migration and/or colony formation in soft agar of melanoma cells, hepatocellular carcinoma cells and breast, prostate, pancreatic and colorectal cancer cell lines [196-198,201,205-207]. It has been reported that the anticarcinogenic effects of these structural analogs of curcumin were mediated, in part, *via *the down-regulation of the expression levels and activities of erbB2, Akt and STAT-3 phosphorylated forms [196-198,205,206]. Importantly, a non-toxic fluorinated curcumin analog, namely EF24, has also been reported to display improved pharmacokinetic profile and bioavailability and greater growth inhibitory and apoptotic effects than those of curcumin on lung, breast and prostate cancer cell lines *in vitro *and animal models *in vivo *(Figure 3) [82,83,86-88,208]. Based on the observations indicating that the transmembrane receptor, tissue factor (TF) for coagulation factor VIIa is aberrantly and abundantly expressed on many cancer cells, a new delivery system has also been developed to specifically target the TF-expressing cancer cells with the curcumin analog, EF24 [209]. This drug delivery system consists of using a complex EF24-linker-Phe-Phe-Arg-mk-factor VIIa, which can associate with TF on the surface of cancer cells and release the cytotoxic agent in the cytoplasm after endocytosis. This EF24 deliver system was more effective at causing a cell cycle arrest on human RPMI-7951 melanoma cells and MDA-MB-231 breast cancer cells than the free EF24 compound [209]. Another study on a series of curcumin analogs has led to the design of a novel synthetic analog containing a pentadieone moiety, designated as compound 23, which was more potent than curcumin at inducing the growth inhibitory activity on MCF-7 and MDA-MB-231 breast cancer cells, and LNCaP and PC3 prostate cancer cell lines while it showed no significant effect on the immortalized but non-malignant MCF-10A mammary epithelial cell line (Figure 3) [187]. Interestingly, a novel non-toxic curcumin analog, namely 5-bis (4-hydroxy-3-methoxybenzylidene)-*N*-methyl-4-piperidine (PAC), also exhibited higher water solubility and stability in blood and greater biodistribution and bioavailability than curcumin [210]. PCA also displayed higher efficiency than curcumin at inducing apoptosis on ER-α negative-MDA-MB-231 breast cancer cells and antitumoral effect on MDA-MB-231 cell xenografts *in vivo *by inhibiting p21^WAP1^, survivin and NF-κB and its downstream effectors, including cyclin D1 and Bcl-2, and activating caspase cascade [210]. Another potent isozazole analog of curcumin was also effective at inducing the cytotoxic effects on hormone-dependent and ER-α expressing MCF-7 breast cancer cells and hormone-independent and multidrug resistant MCF-7R variant which lacks aromatase and ER-α [55].

On the other hand, various chemical analogs of curcumin, including ASC-J9 and its derivatives, have been shown to inhibit prostate cancer cell proliferation by enhancing AR degradation or by acting as the pure AR antagonist [84,204,211]. For instance, the characterization of a series of curcumin analogs, which can function as a 17α-substituted dihydrotestosterone (DHT), has indicated that these compounds display potent anti-androgenic activities superior to current clinical anti-androgenic drug, hydroxyflutamide on AI PC3 and DU145 cells transfected with wild-type AR or mutant LNCaP AR and ARA70 co-activator respectively [204]. Moreover, a chemical hybrid molecule with two bulky side chains, designated as compound 6, which has been derived by a combination of curcumin and β-ionone backbone, was also effective at inhibiting the wild-type or mutant AR activity and induce the cytotoxic effects on AD and AI prostate cancer cell lines *in vitro *[211].

More recently, some chemically stable curcumin derivatives have also been shown to be more effective than free curcumin for eradicating chemoresistant cancer cells with the stem cell-like features from diverse cancer cell lines. For instance, the difluorinated analog of curcumin, 3,4-difluoro-benzo-curcumin, designated as CDF, alone or in combination with 5-fluorouracil and oxaliplatin, was more potent than curcumin at reducing the number of chemoresistant HCT-116 and HT-29 colon cancer cells expressing CD44 and CD166 stem cell-like markers as well as inhibiting the growth and inducing the apoptosis and disintegration of colonospheres *in vitro *(Figure 3) [212]. The anticarcinogenic effects of this difluorinated curcumin analog were mediated through the down-regulation of the expression and/or activity of EGFR, IGF-1R, NF-κB, c-Myc, β-catenin, COX-2 and Bcl-xL signaling components and ABCG2 multidrug transporter combined with an activation of the mitochondrial pro-apoptotic factor Bax [212]. In addition, this difluorinated curcumin analog or CDF was more effective than curcumin at inhibiting the sphere-forming ability and increasing pancreatosphere disintegration of parental and gemcitabine-resistant AsPC-1 and MIAPaCa-2 pancreatic cancer cells *in vitro *[216]. A combination of CDF plus gemcitabine also induced greater tumor growth inhibitory effects on MiaPaCa-2-derived subcutaneous xenografts in severe combined immunodeficient (SCID) mice than curcumin plus gemcitabine through the down-regulation of the NF-κB activity, COX-2 and miR-21 expression and increased expression of phosphatase tensin homolog deleted on chromosome 10 (PTEN) and miR-200 expression [216].

Together, these data suggest that this difluorinated curcumin analog or CDF may be effective at eradicating the total colon or pancreatic cancer cell mass including drug-resistant cancer cells with stem cell-like properties.

#### Novel nanotechnologies and delivery systems of curcumin

Diverse curcumin formulations have been developed with different nanotechnologies consisting of its encapsulation or conjugation with nanoparticles, polymeric micelles or liposomes to improve its stability, bioavailability and specific and sustained delivery into cancer cells and, consequently, its anticarcinogenic effects (Figure 1) [24,89-101,213,214]. For instance, it has been shown that the curcumin encapsulation in biodegradable and biocompatible poly(lactic-co-glycolic acid) (PLGA) nanospheres resulted in an enhanced intracellular uptake of curcumin-loaded polymeric nanospheres and improved cytotoxic effects of curcumin on metastatic LNCaP, PC3 and DU145 prostate cancer cell lines *in vitro *relative to free curcumin, *via *the inhibition of NF-κB activity [24,91,94]. Similarly, a PLGA nanoparticle formulation of curcumin conjugated with a monoclonal antibody specific for ovarian cancer cells also sensibilized the cisplatin-resistant A2780CP ovarian cancer cells to the anti-proliferative and cytotoxic effects induced by cisplatin or radiation *via *the down-regulation of the expression of β-catenin, Bcl-xL and Mcl-1 pro-survival proteins [95]. The complexation of poly-β-cyclodextrin (PCD) and curcumin was also effective at improving the intracellular uptake of curcumin into C4-2, DU145 and PC3 prostate cancer cells and its cytotoxic effects on these cancer cells as compared to free curcumin [93]. Moreover, the cyclodextrin-curcumin complex formulation was more effective than the free curcumin at blocking NF-κB-induced gene expression such as cyclin D1, MMP-9 and VEGF, mediating the anti-inflammatory and anti-proliferative effects on various cancer cell lines and inducing apoptosis in leukemia cells [96]. Moreover, the loading of curcumin into the copolymeric micelles of poly(ethylene oxide)-b-poly(epsilon-caprolactone) (PEO-PCL) has also been shown to be an effective strategy to enhance its solubility, metabolic stability and delivery in diverse cancer cells [92].

In addition, novel curcumin formulations have also been shown to improve the therapeutic effects induced by different chemotherapeutic drugs. For instance, the systemic administration of gemcitabine plus polymeric micelle-encapsulated curcumin formulation displaying higher bioavailability in plasma and tissues as compared to free curcumin, induced greater tumor growth inhibitory and antimetastatic effects than curcumin on pancreatic cancer cells subcutaneously or orthotopically implanted in nude mice *via *an inhibition of NF-kB and its targeted genes [101]. Moreover, the co-administration by oral gavage of liposomal forms of curcumin or resveratrol, prepared by mixing the phytochemical with the liposomal lipid 1, 2-dimyristoyl-rac-glycero-3-phosphocholine, cooperatively reduced the incidence of prostatic adenocarcinoma development in prostate-specific *PTEN *knockout mice as compared to a single liposomal curcumin or resveratrol formulation [89]. It has also been shown that curcumin or resveratrol, alone or in combination, induced the growth inhibitory and apoptotic effects on PTEN-CaP8 prostate cancer cells derived from PTEN-knockout mice model of PC by the down-regulation of the expression levels of pAkt, cyclin D1, the mammalian target of rapamycin (mTOR) and AR proteins [89].

Hence, the use of these novel chemical analogs and nanotechnology-based formulations of curcumin represents a potential alternative strategy of great clinical interest for overcoming the high metabolic instability and poor bioavailability of curcumin, which are among the principal factors limiting its therapeutic effects when administrated orally.

### Concluding remarks

Taken together, these studies carried out in the last ten years have indicated that curcumin may act on multiple oncogenic targets frequently deregulated in cancer cells, including cancer stem/progenitor cells with stem cell-like properties, during the progression of most human aggressive cancers. Despite great interest in using the curcumin as chemopreventive or therapeutic agent, its high metabolic instability and poor systemic bioavailability constitute the major obstacles to its applications in human. Of clinical importance, recent studies have led to the development and validation of novel curcumin formulations with improved pharmacodynamic and pharmacokinetic properties and anticarcinogenic efficacy that offer great promise for overcoming treatment resistance and curing cancer patients at different stages of disease progression. Especially, these novel curcumin formulations could be used to simultaneously target different tumorigenic cascades initiated by different growth factors such as EGFR family members, hedgehog, Wnt/β-catenin and their downstream signaling elements such as PI3K/Akt and NF-κB as well as multidrug resistance transporters that may cooperate for the acquisition of an aggressive behavior by cancer cells during disease progression, treatment resistance and disease relapse.

Selective delivery of curcumin or its synthetic analogs to tumors, alone or in combination with other anticancer drugs, may improve their chemopreventive and chemotherapeutic efficacies against cancer progression and relapse. Novel curcumin formulations may also be used to reverse drug resistance, eradicate the total cancer cell mass and improve the anticarcinogenic efficacy of the current anti-hormonal and chemotherapeutic treatments for patients with various cancers.

Additional structure-activity relationship studies of curcumin and characterization of the pharmacokinetic and anticarcinogenic proprieties of novelty identified synthetic analogs and nanotechnology-based formulations of curcumin, however, are essential to optimize their physicochemical and anticancer properties and selective delivery to tumor before they can be safely used in the clinics. More particularly, the selective targeting of nanoparticle-encapsulated curcumin formulations to tumors could be further improved by their conjugation to ligand or monoclonal antibodies (Figure 1). Future pre-clinical studies are also required to more precisely establish the mechanisms of absorption, distribution, mode of action and antitumoral, anti-metastatic and chemosensibilizing effects of curcumin and its derivatives on diverse animal models *in vivo*. Particularly, it will be important to determine the therapeutic benefit and chemosensibilizing effects of combining low doses of curcumin and its derivatives with other nutraceuticals and/or chemotherapeutic drugs used in the clinics. Furthermore, the results from randomized, double blind and placebo-controlled clinical trials carried out with more cancer patients and after a long-term treatment with curcumin and its derivatives, alone or in combination with the current therapies, are also necessary to confirm their bioavailability, chemopreventive and therapeutic effects, drug interactions and potential synergy of action on distinct cancer subtypes.

Thus, these future investigations should lead to more chemically stable and effective curcumin formulations that could be used as dietary substances, in safe conditions for cancer prevention. These therapeutic agents also could be used, alone or in combination with current cancer therapies, for treating the patients diagnosed at the early and late stages with diverse types of aggressive and recurrent cancers, and thereby prevent disease relapse and the death of cancer patients.

## Abbreviations

ABC: ATP-binding cassette; AD: androgen-dependent; AI: androgen-independent; AP-1, activator protein-1; APC: adenomatous polyposis coli; AR: androgen receptor; cIAPs: cellular inhibitor of apoptosis proteins; COX-2: clyooxygenase-2; Cyt: cytochrome C; Dvd: disheveled; EGFR: epidermal growth factor receptor; ER-α: estrogen receptor-α; Fzd: frizzled receptor; IGF-1R: insulin-like growth factor type-1 receptor; IL-6: interleukin-6; LRP: low density lipoprotein receptor-related protein; MMPs: matrix metalloproteinases; NF-κB: nuclear factor-kappa B; PARP: poly(ADP-ribose) polymerase; PI3K: phosphatidylinositol-3'-kinase; PTCH: patched receptor; PTEN: phosphatase tensin homolog deleted on chromosome 10; RTK: receptor tyrosine kinase; SCID: severe combined immunodeficient; SHH: sonic hedgehog; STATs: signal transducers and activators of transcription; uPA: urokinase-like plasminogen activator; VEGF: vascular endothelial growth factor; Wnt: wingless ligand.

## Competing interests

The authors declare that they have no competing interests.

## Authors' contributions

MM searched and reviewed the literature and wrote the manuscript. SKB reviewed the literature and revised the manuscript. Both authors read and approved the final version of manuscript.

## Supplementary Material

Additional file 1 **Clinical trials on the evaluation of the safety, and chemopreventive and chemotherapeutic effects of curcumin alone or in combination therapy**. Clinical trial data on the safety, chemopreventive and chemotherapeutic effects of curcumin alone or in combination therapy. Click here for file
