# Supplementary material for: Potential applications of curcumin and its novel synthetic analogs and nanotechnology-based formulations in cancer prevention and therapy
Source: Chin Med. 2011 Aug 23;6:31. doi: 10.1186/1749-8546-6-31 (PMC3177878; doi:10.1186/1749-8546-6-31)
Supplement: Additional file 1 — Clinical trials on the evaluation of the safety, and chemopreventive and chemotherapeutic effects of curcumin alone or in combination therapy. Clinical trial data on the safety, chemopreventive and chemotherapeutic effects of curcumin alone or in combination therapy. [file 1749-8546-6-31-S1.DOC]

**Clinical trials on the evaluation of the safety, and chemopreventive and chemotherapeutic effects of curcumin alone or in combination therapy**

| **Design and objectives of the trial** | **Number of patients** | **Tested doses of curcumin alone or in combination with another drug** | **Tumor response and outcomes of the patients** | **Toxicity and side effects** | **Ref** |
| --- | --- | --- | --- | --- | --- |
| A phase I dose-escalation trial to determine the safety, efficacy and pharmacokinetics of curcumin in cancer chemoprevention | 25 patients with high-risk or pre-malignant lesions | Different doses of oral curcumin varying from 500 to 12,000 mg/day were tested for 3 months | Histological improvement of high-risk or precancerous lesions in some patients | Good tolerability and no toxicity of curcumin at doses of 8000 mg/day or lower | [71] |
| A dose-escalation pilot study to establish the pharmacodynamic and pharmacokinetic profiles of curcumin in patients with advanced colorectal cancer refractory to standard chemotherapies | 15 patients with advanced colorectal cancer refractory to standard chemotherapies exhibiting PD before recruitment | 5 different doses of curcumin varying from 400 to 2200 mg/day of *Curcuma* extract containing 36-180 mg of curcumin were tested. Each patient received a same dose of oral curcumin daily for up to 4 months. | Radiological assessment of target lesions had indicated that 5 patients had SD after treatment with oral dose of curcumin for 2-4 months. | Curcumin was well-tolerated and no DLT has been observed at doses tested. Only two patients experienced mild diarrhea and one nausea. | [72] |
| A phase I dose-escalation study to establish the toxicity and pharmacokinetic and pharmacodynamic parameters of curcumin in patients with advanced colorectal cancer refractory to standard chemotherapies | 15 patients with advanced colorectal cancer refractory to standard chemotherapies. All of the patients enrolled in this study exhibited a DP before recruitment. | 4 different doses of curcumin varying from 450 to 3600 mg were tested. Each patient received a same dose of oral curcumin daily for up to 4 months | Response assessment was made with *WHO response criteria. 1 patient had a SD after treatment with curcumin for up to 4 months while another patient showed a SD after 2 months that has subsequently progressed. | No DLT has been observed at doses of curcumin tested | [73] |
| A phase II trial to evaluate the clinical biological effect of curcumin against advanced pancreatic cancer | 25 pancreatic cancer patients including 24 evaluable for toxicity and 21 for tumor response | Patients received 8000 mg of oral curcumin by month daily until disease progression with restaging every 2 months | Response assessment was made according to **RECIST criteria. One patient had a SD for more than 18 months and another patient exhibited a brief, but marked, tumor regression | No toxicity has been noticed at the dose of curcumin tested | [77] |
| A phase I/II study to establish the safety and feasibility to use a combination of curcumin plus gemcitabine for treating pancreatic cancer patients refractory to gemcitabine-based chemotherapy | 21 pancreatic cancer patients who showed DP during gemcitabine-based chemotherapy | Patients received a dose of 8000 mg/day of oral curcumin extract containing 73% curcumin, 22% demethoxycurcumin and 4% bisdemethoxycurcumin. Moreover, 19 patients received gemcitabine/S-1 and two patients received gemcitabine in addition to oral curcumin. | Response assessment was made according to **RECIST criteria. 5 patients showed SD and several patients had an improvement on cancer- and chemotherapy-related symptoms such as fatigue, pain and constipation. | Grades 3/4 hematological and non-hematological adverse events associated with gemcitabine as well as 4 cases of grade 1 diarrhea with no DLT have been observed | [81] |
| A phase I trial to evaluate the activity and feasibility of gemcitabine plus curcumin in patients with previously untreated locally advanced or metastatic pancreatic cancer | 17 patients including 11 evaluable patients with advanced pancreatic cancer | 8000 mg/day of oral curcumin by month plus gemcitabine 1000 mg/m^2^ IV weekly ×3 out of 4 weeks | Response assessment was made by using **RECIST criteria. 1 patients had a PR, 4 SD and six had tumor progression. Time to tumor progression was 1-12 months (median 2.5), and overall survival was 1-24 months (median 5). | One patient with grade 2 neutropenia and another with grade 1 thrombocytopenia. | [178] |
| A phase I trial to estimate the maximal tolerated dose and efficacy of a combination of dose-escalating curcumin and standard dose of docetaxel for treating the patients with advanced and metastatic breast cancer | 14 patients with advanced and metastatic breast cancer including 13 evaluable patients for toxicity and 9 for tumor response | Each patient received a same dose of oral curcumin varing from 500 to 8000 mg/day for 7 consecutive days from day -4 to day +2 during six cycles of treatment. Moreover, each patient received an intravenous infusion of 75 or 100 mg/m^2^ of docetaxel on day 1 every 3 weeks for six cycles. | Response assessment was made according to **RECIST criteria. 5 patients showed PR and 3 SD. Moreover, 1 patient had evaluable bone lesions which were stable after 6 cycles of treatments. | Hematological toxicity after docetaxel treatment including one patient had grade 4 neutropenia with DLT. Moreover, two patients experienced grade 3 diarrhea with DLTs. | [177] |

**Abbreviations:** DLT, dose-limiting toxicity; PR, partial response; DP, disease progression; SD, stable disease. Measurements for the solid tumor response of cancer patients to tested agents were made by using *Standard World Health Organization (WHO) Solid Tumor Response Criteria or **Response Evaluation Criteria in Solid Tumors Group (RECIST) [179].
